# Floral visitors of sesame (*Sesamum indicum* L.): Elucidating their nectar-robbing behaviour and impacts on the plant reproduction

**DOI:** 10.1371/journal.pone.0300398

**Published:** 2024-04-18

**Authors:** Ujjwal Layek, Trisha Bhandari, Alokesh Das, Prakash Karmakar

**Affiliations:** 1 Department of Botany, Rampurhat College, Birbhum, India; 2 Department of Botany & Forestry, Vidyasagar University, Midnapore, India; Indian Institute of Science, INDIA

## Abstract

Nectar robbing is common in angiosperms, especially in long tubular flowers or flowers with spurs that keep nectar out of reach of visitors. However, the robbing behaviour of bees is less understood. Here, we studied the sesame visitors, their robbing behaviour, and the impacts of robbing on plant reproductive fitness. Diverse insect species (primarily members of Hymenoptera) visited sesame flowers. The most effective pollinators were *Amegilla zonata*, *Apis cerana*, *Apis dorsata*, *Apis florea*, *Ceratina binghami*, *Halictus acrocephalus* and *Xylocopa amethystina*. Almost all visitors with variable percentages revealed the nectar-robbing phenomenon. Robbing activity depended on a complex of multiple attributes, including the visitor’s body size, the corolla tube length, the availability and accessibility of nectar, and the resource-collecting task allocation of bees. Robbing activity varied according to flower-visiting species, flowering period and daytime. Robbing was comparatively higher in the late flowering period at 10.00–14.00 h. In the case of robbing visits, flower handling time was lower, and the visitation rate remained higher than non-robbing visits. Robbing visits did not significantly affect fruit and seed sets of sesame. Therefore, we can interpret the nectar-robbing interactions on sesame as commensal, with pollinators benefitting without altering the plant’s reproductive fitness.

## Introduction

Plants and floral visitors rely on one another for reproduction and food resources. One of the most enduring research problems in reproductive biology is understanding how flowering plants invest resources into reproduction and regulate the dynamics of plant-pollinator interaction. Floral visitors show different strategies for resource collection, including legitimate (if they touch the reproductive parts of flowers) and illegitimate (if they do not touch the reproductive parts of flowers; serving as nectar robbers, nectar thieves, and pollen thieves) types of visitation [1–3].

In angiosperms, nectar robbing is common, mostly in long tubular flowers or flowers with spurs where nectar is kept out of reach of visitors [4,5]. Nectar robbers pierce the perianth or corolla tube of flowers to access the nectar, either by themselves or by other robbers [6–8]. The flowers of some plant species do not rob; some (e.g. *Rouvolfia serpentina* (L.) Benth. Ex Kurz: author’s observation) are occasionally robbed; and a few (mainly having long tube corolla, e.g. *Tirpitzia sinensis* Hallier f. [9]) are more likely to be robbed. The frequency of robbing visits may depend on several attributes, like the abundance of flowers, corolla length, floral resource accessibility, and temporal and spatial change in the abundance of robbers [10–12]. Visitor morphometry (e.g., body size and proboscis length) and resource-collecting behaviour may also affect their robbing activity, but this remains unclear. Floral visitors’ robbing behaviour has varying effects on plant reproductive success, ranging from zero to high impact and might be negative or positive to plant fitness [13,14]. As a result, nectar robbers are thought to be one of the selective forces driving plant evolution, shaping population structure and community dynamics [15,16].

Despite the importance of studies on nectar-robbing behaviour in understanding the evolution and stability of plant-pollinator interactions, only a few research approaches have attempted to elucidate the ecological complexity regarding plant-pollinator-larcenist interactions at community or system levels [17–19]. Many nectar-robbing studies have taken the plant’s perspective, testing the effects of nectar-robbing on the plant’s reproductive success [19,20]. However, knowledge about the nectar-robbing behaviours of floral visitors is still too limited, fragmented, and localised. Therefore, we designed the present work to determine the nectar-robbing behaviour of floral visitors on sesame (*Sesamum indicum* L.) flowers and the impacts of robbing on the plant’s reproduction. We aim to answer the following research questions: (1) What are the floral visitors, robbers and pollinators of sesame? (2) Do the visitation rate and flower handling time differ between robbing and non-robbing visits and vary diurnally? (3) What flower and bee traits facilitate robbing activity in flowers? (4) What is the impact of robbing activities on plant reproduction?

## Materials and methods

### Plant species

We conducted experiments on an oilseed crop, sesame (*Sesamum indicum* L.) var. Rama belongs to the plant family Pedaliaceae. The plant is herbaceous and less branched. The flowering period was from April to June. Flowers are borne on dichasium inflorescences and open early in the morning (5.00–6.00 h). The abscission of the corolla tube starts at 17.00 h and continues onwards. During peak blooming time, about three flowers opened per plant daily. Zygomorphic flowers are whitish with violet marking, pendulous corolla tube (35–50 mm in length), stamens four, epipetalous, didynamous; gynoecium is bicarpelar, with bilocular ovary, style filiform, and bifid stigma. The average nectar production per flower (dry weight) varies from 0.21–0.54 mg [21]. The plant is self-compatible and, to some extent, auto-pollinate; however, insect visitation enhances fruit and seed sets [22].

### Experimental site

Most of the work was conducted in open fields of farmers at Jenadihi (23.4468° N and 87.0449° E) village of Bankura district, West Bengal, India, during 2022–2023. Additionally, the effect of legitimate and robbing visits on plant reproduction was studied in two fields (5 m × 3 m) at Vidyasagar University campus (22.4320° N and 87.2979° E), Paschim Medinipur district, West Bengal. At Jenadihi village, the selected cultivated zone comprises many sesame fields, and we collected data from ten fields (situated at a single agricultural zone) of different sizes (length: ranges from 8.26–15.42 m; breadth: ranges from 6.27–10.38 m). Among these ten fields, some sesame fields are situated closely side by side, sharing common ridges. A few uncultivated fields are also there within the selected agricultural zone. During the blooming period of sesame, the study areas prevailed in a hot, dry summer season with a maximum day temperature of 44°C.

### Floral visitors

We surveyed the sesame fields in the daytime, which segregated into six timeslots (6.00–8.00 h, 8.00–10.00 h, 10.00–12.00 h, 12.00–14.00 h, 14.00–16.00 h and 16.00–18.00 h). We recorded the visitor’s abundance (i.e., number of visitors per m^2^ area per 5 minutes) on sesame fields at each timeslot (N = 180 observations, n = 30 observations per timeslot; the duration of each observation was 5 min). We identified the flower-visiting species in the fields or captured them and sent them to entomologists (at the Zoological Survey of India, Kolkata, West Bengal) for identification. We have taken photographs of some insect visitors.

The relative abundance (RA) of each flower-visiting species was calculated as follows:

$$RA\;(\%)=\frac{ni}{N}\times 100$$

where n*i* is the number of encountered individuals of the insect species *i* and N is the total number of encountered individuals of all flower-visiting species.

We estimated the single-visit pollination efficiency index (PE*i*) of abundant visitors by using the method of Spears [23], based on seed sets in three treatments- (i) open-pollination, i.e. unrestricted visitation, (ii) visitor-exclusion treatment, and (iii) single visit. We conducted these three experiments on different plants grown in a single field. For the visitor-exclusion treatment, we marked a few matured flower buds (n = 10) within a small patch (containing 5–8 plants) and covered the patch with a nylon net in the late afternoon. We observed the marked flowers (N = 10 × 10 flowers; 10 sampling days) blooming from the selected buds. For the single-visit experiment, we covered a small area (1 m × 1 m) like the visitor-exclusion experiment. During the peak foraging time of visitors (about 9.00 h), we uncovered the flowering patch and watched the virgin flowers for the first visit by one of the focal bees. Once visited, we tagged the flowers to indicate the treatment and immediately re-enclosed them by netting to prevent further visitation. For the open-pollination treatment, we marked ten flowers in the morning on a sampling day (N = 10 × 10 flowers; 10 sampling days). We estimated fruit set percentages for these treatments. We also recorded seed sets per flower (N = 30 flowers for open and N = 30 flowers for pollinator-exclusion treatments; N = 27–53 flowers for the single visit of an insect species) after 10–15 days of flower opening. Then, we calculated the PE*i* as follows:

$$PEi=\frac{Pi-Z}{U-Z}$$

Where P*i* is the mean number of seeds per flower resulting from a single visit of species *i*; Z is the mean number of seeds per flower in the visitor-exclusion treatment; and U is the mean number of seeds per flower resulting from unrestricted visitation.

We recorded primary robbers (the individuals who made a hole in a corolla tube and robbed the unrob flowers) and secondary robbers (individuals who obtained nectar via the hole made by the primary robbers). For that, we covered a small portion of the sesame field (1 m^2^ area) in the late afternoon. On the next day, we uncovered the flowering patch at peak visitation time (about 9.00 h). We observed the visitation patterns (mainly, we focused on robbing visits) of the visitors on these flowers (n = 10 flowers on each sampling day; 4 sampling days; we selected 1–2 flowers per plant). For the robbing visits, we carefully watched whether the visiting species pierced and made a hole in the corolla tube to steal nectar (i.e., treated as primary robbers) or obtained nectar through a hole made by other primary robbers.

We recorded the flower visitation rate (i.e., the number of flowers visited in a 1-minute duration) and the flower handling time (i.e., the amount of time spent per visit on a flower) for sesame visitors [24]. To estimate the visitation rate, we started a stopwatch when a focal visitor came in contact with a flower (to collect floral resources), followed the flower-visiting individual for 1 minute and counted the number of flowers visited in this time span. For an insect species, we conducted 120 observations (N = 20 × 6; 20 observations per timeslot, 6 timeslots) for non-robbing visits and 120 observations (N = 20 × 6) for robbing visits (as per their visiting pattern). We also used a stopwatch to record the handling time [N = 120 observations for non-robbing visits (20 observations per timeslot, 6 timeslots), N = 120 observations for robbing visits (20 observations per timeslot, 6 timeslots) for a flower-visiting species], starting when a visitor came in contact with a flower and continued until the visitor left the flower.

We documented the type of floral resources (nectar, pollen or both) collected by a flower-visiting species. On close observations (without touching or capturing the visitors) on a flower-visiting individual, we recorded the floral resources collected by the visitors during 8.00–10.00 h (i.e., the period when both nectar and pollen were available for the visitors). For abundant visitor species, we took 100 observations for an insect species. However, the sample sizes were lower for the less abundant visitors.

To estimate the percentage of robbing visits, we randomly observed visits for the insect species on several sesame fields at the selected agricultural zone. For more abundant flower-visiting species (e.g., *Apis cerana*, *Apis dorsata*, *Apis florea*, *Ceratina binghami* and *Halictus acrocephalus*), we took data from three blooming periods viz. early (in early April), middle (i.e., peak flowering period, mid-April–mid-May) and late (in June). For each of the three periods, we took 60 observing samples (n = 10 × 6; 10 samples per timeslot, 6 timeslots) and each sample comprised 20 encountered visits for an insect species. For other insect species, we collected data during peak flowering time only (observing sample size for an insect species, n = 10 × 6; 10 samples per timeslot, 6 timeslots, and each sample comprised 20 encountered visits). Then, we calculated the percentages of robbing visits, considering the number of robbing visits and the total number of visits encountered for an insect species.

We sat near a flower patch and recorded the number of robbing visits successively done (i.e., in an uninterrupted manner) by the visitors. When robbing visits were interrupted by a non-robbing visit, we stopped counting on this. We randomly selected an individual of focus insects visiting the flowers as a robber, followed and counted successive robbing visits of the individual up to 31 visits (the counting number is not too small or not too large to measure for an individual as they move through the crop field and left the field after fully loaded). In this way, we took 50 counts for each timeslot for each dominant visitor species (data came from several individuals of a flower-visiting species). Then, we calculated the percentages of their robbing visits fall into each of four groups (i.e., <10, 10–20, >20–30, and >30; these are the number of successive robbing visits).

### Drivers of robbing activity

#### Visitor’s body size and robbing activity

To measure body size (body length: considering the length of head, thorax and abdomen but excluding antennae; intertegular length: the distance between the tegulae), we caught flower-visiting individuals (generally three individuals for an insect species) for each species using a hand-held entomological net. We do not collect data about the foraging activity of visitors on the sampling days employed for catching insects (4 days in peak flowering time; 8.00–12.00 h in each sampling day) as caught insects may influence the foraging activity of visitors for some time. After being caught, we collected the individuals in a 10–30 mL glass vial (depending on the size of the insects) and preserved them with 70% ethanol. The body length (for all 21 insect species) and intertegular span (for 18 hymenopteran members) of the preserved samples were measured using a stereo microscope (Zeiss Stemi 508 trinocular microscope). Then, we estimated the relationship between visitor’s body size (considering the average values for body length and intertegular distance for an insect species) and percentages of robbing visits.

#### Corolla tube length and robbing activity

Here, we considered three types of flowers─ (1) normal flowers, i.e. regarded as control set (S1A Fig; N = 100 flowers, 10 flowers on a sampling day, 10 sampling days), (2) shortened corolla (about 20 mm in length) without leaving any landing space (S1B Fig; N = 100 flowers, 10 flowers on a sampling day), and (3) shortened corolla (about 20 mm in length for tubular region) with landing space (S1C Fig; N = 100 flowers, 10 flowers on a sampling day, 10 sampling days). One to two flowers were chosen from an individual plant, and the plants were randomly distributed in a field. For the third experiment, we shortened the length of the tubular region, leaving the landing space for visitors. The visitors can forage on these flowers using the landing space, and their proboscis could easily reach the nectar source as we reduced the tube length. We shortened the corolla tube length by cutting the corolla (100 flowers for each of the two categories) in the early morning (at 4.00–5.00 h) during the peak flowering period without altering floral resource content. Then, we recorded the visitation pattern of floral visitors (number of visits received and types of visits, i.e., robbing and non-robbing) on each selected flower for a 10-minute observation period (data were taken during peak visiting time, about 9.00–10.00 h).

#### Scarred corolla tube and robbing activity

**Fig 1:**
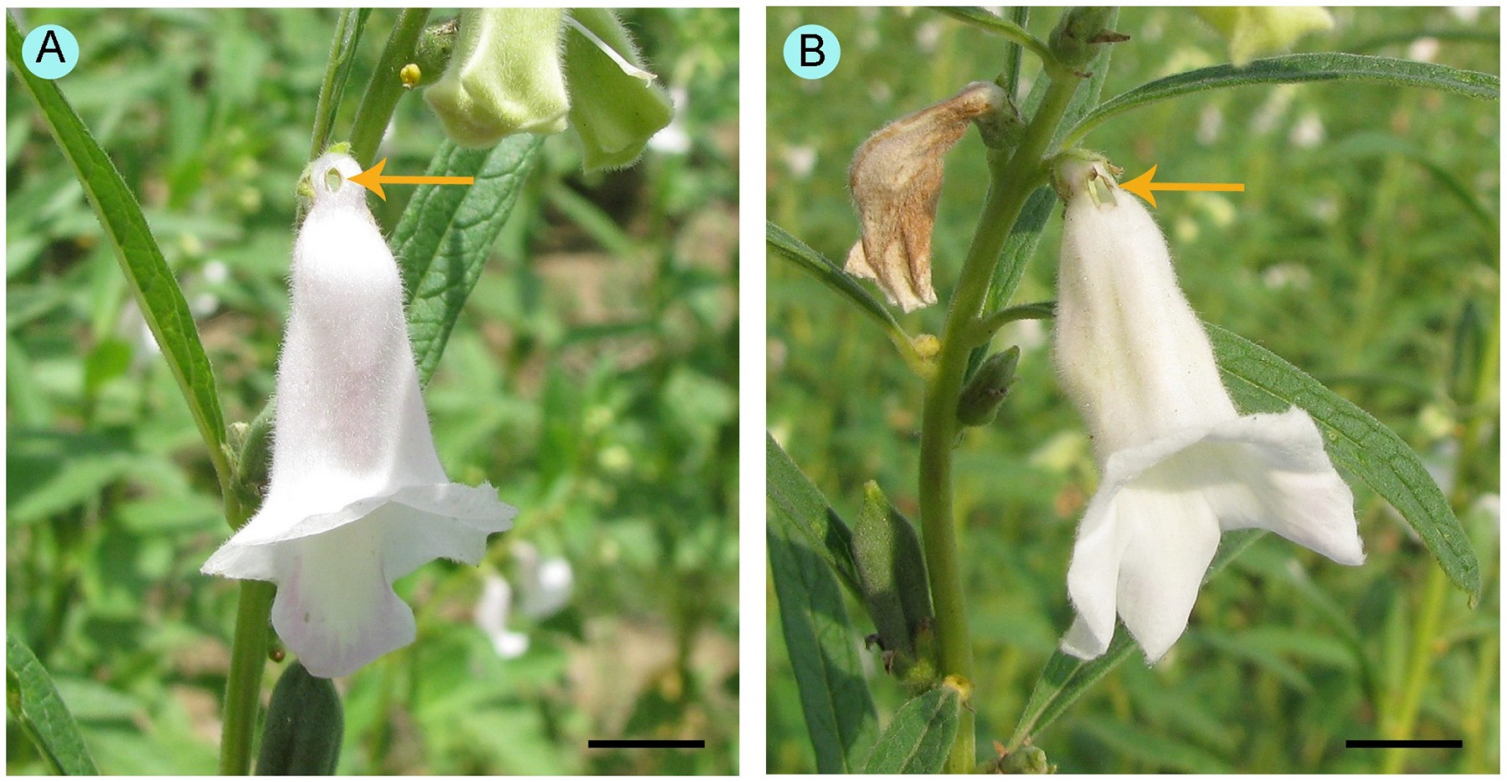
Flowers showing scars on corollae tubes made by (A) floral visitors and (B) artificially. Scale bars = 10 mm.

Several robbing visits on sesame flowers made scars (those more prominently appeared from 11.00 h onwards) at the base of the corollae tubes (Fig 1A). To determine the effect of these scars on robbing activity, we selected a few flowers (N = 100, n = 10 on a sampling day) and artificially produced a scar at the base of the corolla tube (Fig 1B) using a needle during the early morning (5.00–6.00 h). Then, we observed the visitation patterns of floral visitors during the early visiting time (6.00–7.00 h) when the normal (i.e., control) flowers were without scars, and we compared them with the visitation patterns on normal flowers.

#### Floral rewards and robbing activity

Here, we recorded observations on four types of flowers─ (1) normal flowers (i.e., control set), (2) pollen-less flowers with normal nectar quantity, (3) pollen-less with higher nectar quantity (higher in compared to normal flowers i.e., control set), and (4) flowers with higher nectar and pollen quantities (higher in compared to normal flowers). To make a flower pollen-less with normal nectar, we simply removed anthers of some flowers (N = 100, n = 10 flowers on a sampling day) by forceps in the morning. Then, we observed the visitation pattern of floral visitors and compared it with normal flowers (data were taken at 7.00–8.00 h when normal flowers have a significant amount of pollen content). To make a flower pollen-less with higher nectar quantity, we removed anthers of flowers (N = 100, n = 10 flowers on a sampling day) of closely situated plants (5–8) in the early morning (4.00–5.00 h). Then, we covered these plants with a nylon net to restrict the visitation of floral visitors. At peak foraging time (about 9.00–10.00 h), we uncovered the plants by removing the net and observed the visitation pattern of floral visitors for 10 minutes. We also covered a few plants (5–8) with a nylon net during the early morning (4.00–5.00 h) to make the bagged flowers with higher nectar and pollen content than normal ones. At peak foraging time, we uncovered the plants and recorded visitation patterns on the selected flowers (N = 100, n = 10 flowers on a sampling day).

#### Resource collecting task allocation and robbing activity

Flower-visiting individuals may be specialized nectar foragers (those who collect only nectar), specialized pollen foragers (those who collect only pollen grains) or mixed foragers (those who collect both nectar and pollen grains) [3]. These foraging categories (here, specialized nectar foragers and mixed foragers) may have different robbing activities, which we assessed on honeybees. For that, we randomly choose robbers (N = 200, n = 20 per sampling day for each honeybee species) and non-robbers (N = 200, n = 20 per sampling day for each species) at 8.00–10.00 h (when nectar and pollen are available for visitors). After choosing a robber and a non-robber, we carefully observed the visitations of an individual forager and determined whether it was specialized nectar or mixed forager. We recognized a specialized nectar forager by the following observations─ (i) the visitor reached the base of the corolla tube, (ii) the legs moved less, and (iii) there was no grooming or brassing after leaving a flower. We identified a mixed forager by the following observations─ (i) the visitor reached the base of the corolla tube to collect nectar, (ii) the legs moved more and occurred near the opening end of the tube where the anthers are located, (iii) after leaving the flowers, the forager groomed herself and brushed pollen sticking to her body towards her hind legs, and (iv) there may be an observable amount of pollen loads on the corbiculae. Then, we compared the percentages of specialized nectar foragers and mixed foragers between robbers and non-robbers categories.

### Impact of legitimate and illegitimate visits on plant reproduction

We recorded fruit and seed sets for open field conditions, pollinator-exclusion treatment and single legitimate visit experiments (already mentioned for the PE*i* estimation). We also recorded the fruit and seed sets for single illegitimate (i.e., robbing) visits of some pollinators and multiple visits [three categories─ (i) multiple legitimate visits, (ii) initial legitimate visit, followed by mixed types of visits, and (iii) initial nectar-robbing visit, followed by mixed types of visits] of pollinators. We netted a small area containing 15–20 plants in the late afternoon for single illegitimate visit and multiple-visit experiments. The next morning, we uncovered the plants to receive a visit from the floral visitors. In the case of the single illegitimate visit experiment, after getting an illegitimate visit to a flower (n ≥ 20 flowers for each dominant insect species), we netted the flowers (to restrict further visitation of visitors) until the senescence of the corolla and stigma. In the case of the multiple-visit experiments, we uncovered the plants for receiving multiple visits for a brief observable period (8.00–10.00 h). We marked and labelled the flowers (n = 30–52 flowers for each treatment) that received one of the three types of multiple visits (mentioned above). After the observation period, we again covered the plants with a nylon net.

### Data analysis

Descriptive data analyses were carried out to get the mean and standard deviation. We used the ‘Shapiro-Wilk’ tests to check whether the data was normally distributed. To estimate the relationship between visitor’s body size (body length and intertegular distance) and percentages of robbing visits, we followed Spearman’s correlation method. We carried out an independent t-test to compare the means of two groups (e.g., flower visitation rate between non-robbing and robbing visitation; the amount of time spent on non-robbing and robbing visitation; visitor’s abundance and robbing percentages of control, and pollen-less flowers; visitor’s abundance and robbing percentages between the control and the artificially scarred flowers; fruit and seed sets between open and bagged flowers, legitimate and robbed flowers). To compare means of more than two groups (e.g., daytime wise visitor’s abundance; visitor’s abundance and robbing percentages of control, flowers with high nectar and pollen, pollen less flowers with high nectar; visitor’s abundance and robbing percentages of control, short-tube corolla with landing space and short-tube corolla without landing space; daytime wise flower visitation rate; flowering period wise and daytime wise robbing visits), data were analyzed using a parametric test, One-way ANOVA. If the obtained *p*-value was significant, we conducted Duncan’s multiple range test (DMRT) to evaluate the significant difference among the mean values. We used a generalized linear model (GLM) to examine whether fruit and seed sets differed among the three multiple-visit experiments. A gamma distribution and a logit link function were employed in the models. The pollination treatments (i.e., three types of multiple visits) constituted the fixed factors, while the mean fruit set (%) and seeds per flower were the dependent variables in the models. In our statistical analyses, *p* ≤ 0.05 was considered statistically significant. We conducted the statistical analyses using SPSS (ver. 25.0) statistical packages.

## Results

### Floral visitors of sesame

**Fig 2:**
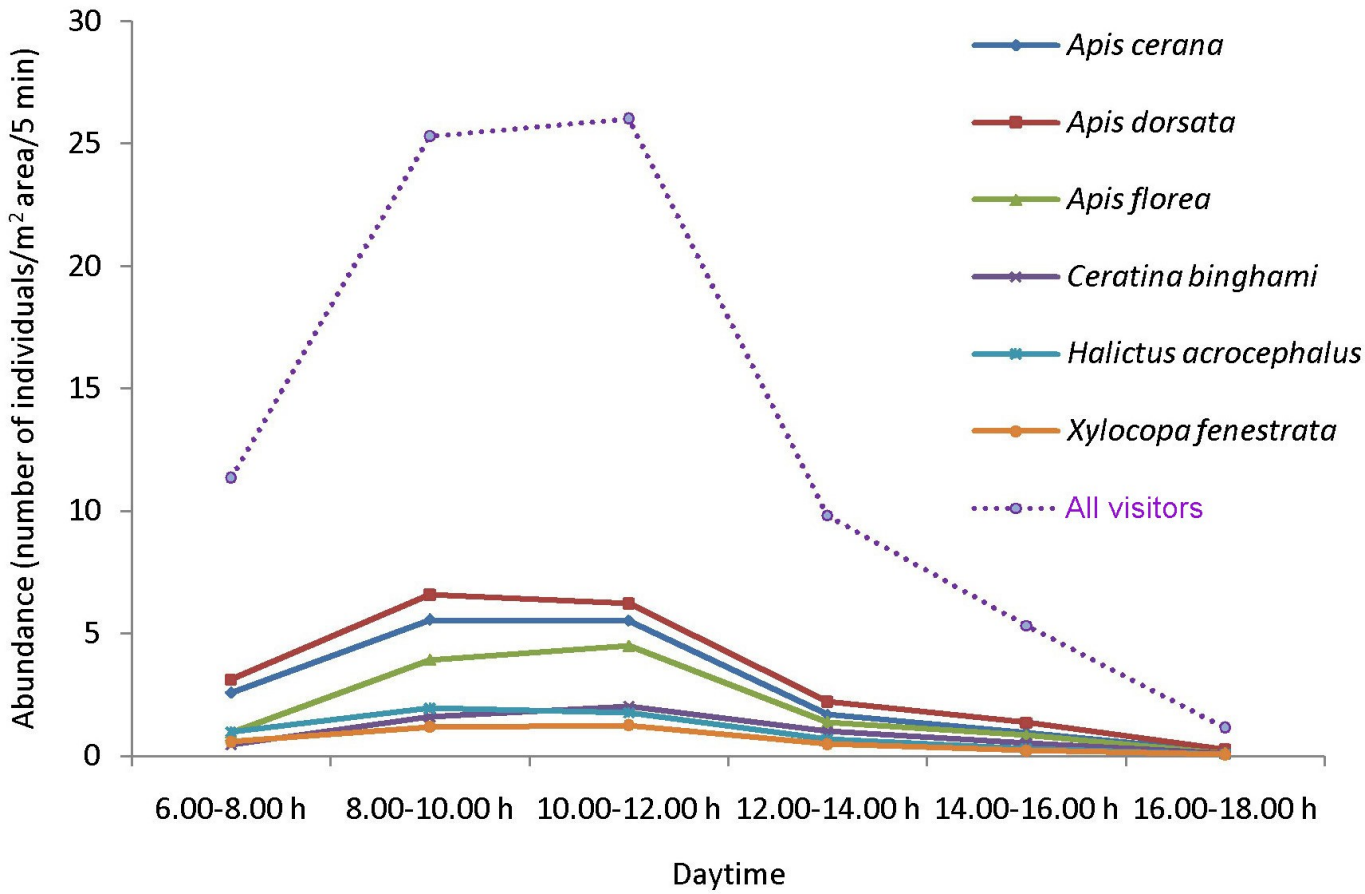
Daytime wise abundance of floral visitors of sesame.

Twenty-one insect species were recorded as floral visitors of sesame in West Bengal, India (Table 1). Among them, 18 species belong to Hymenoptera, two to Lepidoptera and one to Hemiptera. Most abundant visitors were *Apis dorsata* (relative abundance 25.09%), *Apis cerana* (relative abundance 20.92%), *Apis florea* (relative abundance 14.93%), *Halictus acrocephalus* (relative abundance 7.34%), *Ceratina binghami* (relative abundance 7.25%) and *Xylocopa fenestrata* (relative abundance 4.89%). In the daytime (6.00–18.00 h), the average abundance of flower-visiting insects was 13.17 ± 10.71 visitors/m^2^ area/5 min (mean ± SD, n = 180). However, the abundance of floral visitors significantly varied according to daytime (*F*_5, 174_ = 122.14, *p* < 0.001). A higher abundance of visitors was recorded during 8.00–12.00 h (8.00–10.00 h: 25.30 ± 8.47 visitors/m^2^ area/5 min; 10.00–12.00 h: 26.03 ± 7.27 visitors/m^2^ area/5 min) and lower abundance in the late-afternoon (Fig 2).

Most floral visitors collected both nectar and pollen grains from sesame flowers. While a few visitors (e.g., *Xylocopa aestuans*, *Xylocopa fenestrata*, *Xylocopa latipes*, moths and wasps) collected only nectar from sesame flowers. The members of Hymenoptera (excluding *Chalybion bengalense*, *Polistes tenebricosus*, *Xylocopa aestuans*, *Xylocopa fenestrata* and *Xylocopa latipes*) visited sesame flowers legitimately. All these provided pollination services to the plant species. Regarding the single-visit pollination efficiency index, relative abundance and visitation rate of the visitors, the most effective pollinators were *Amegilla zonata*, *Apis cerana*, *Apis dorsata*, *Apis florea*, *Ceratina binghami*, *Halictus* acrocephalus and *Xylocopa amethystina*.

**Table 1:** Floral visitors of sesame (*Sesamum indicum*) in West Bengal, India.

Floral visitors	Relative abundance	Visitation rate (flowers/min)	Flower handling time (sec)	Visitation pattern	PE*i*	Robbing visits (%)	Floral resource
Hemiptera							
*Graptostethus servus*	1.22	-	-	SR	-	100	N
Hymenoptera							
*Amegilla zonata*	3.63	14.60 ± 3.34	2.67 ± 0.90	LV, PR, SR	0.48	13.25 ± 9.82	N + P
*Apis cerana*	20.92	8.37 ± 3.23	4.96 ± 2.54	LV, PR, SR	0.49	23.42 ± 14.45	N + P
*Apis dorsata*	25.09	8.41 ± 2.92	4.95 ± 2.35	LV, PR, SR	0.59	26.08 ± 15.52	N + P
*Apis florea*	14.93	7.06 ± 2.77	5.27 ± 2.51	LV, PR, SR	0.30	25.17 ± 16.34	N + P
*Ceratina binghami*	7.25	3.89 ± 1.79	11.33 ± 7.24	LV, SR	0.27	17.33 ± 12.84	N + P
*Ceratina compacta*	2.07	3.38 ± 1.59	12.54 ± 8.07	LV, SR	0.25	7.83 ± 6.34	N + P
*Chalybion bengalense*	0.55	2.73 ± 0.90	4.03 ± 1.44	PR, SR	-	100	N
*Halictus acrocephalus*	7.34	4.32 ± 1.79	18.74 ± 17.52	LV, PR, SR	0.44	24.58 ± 16.11	N + P
*Megachile monticola*	0.55	8.63 ± 3.16	4.78 ± 2.15	LV, SR	0.53	4.58 ± 4.90	N + P
*Polistes tenebricosus*	0.30	3.22 ± 0.67	4.08 ± 1.48	PR, SR	-	100	N
*Pseudapis oxybeloides*	1.27	4.56 ± 1.79	17.28 ± 15.94	LV, PR, SR	0.41	25.08 ± 17.45	N + P
*Scolia soror*	1.94	4.13 ± 1.05	4.32 ± 1.48	LV, PR, SR	-	92.75 ± 6.79	N
*Tetragonula iridipennis*	1.05	2.60 ± 0.95	37.57 ± 12.98	LV	0.22	0	N + P
*Thyreus nitidulus*	0.84	8.11 ± 3.12	4.69 ± 2.20	LV, PR, SR	0.43	14.75 ± 9.41	N + P
*Xylocopa aestuans*	0.72	12.08 ± 3.28	2.87 ± 0.79	PR, SR	-	100	N
*Xylocopa amethystina*	3.37	15.04 ± 3.42	2.89 ± 0.86	LV, PR, SR	0.58	3.92 ± 4.33	N + P
*Xylocopa fenestrata*	4.89	16.93 ± 4.00	2.59 ± 0.87	PR, SR	-	100	N
*Xylocopa latipes*	1.73	16.22 ± 3.96	2.76 ± 0.82	PR, SR	-	100	N
Lepidoptera							
*Eretmocera impactella*	0.17	-	-	SR	-	100	N
*Utetheisa pulchella*	0.17	-	-	SR	-	100	N

Visitation pattern: LV- legitimate visit, PR- primary robber, SR- secondary robber; PE*i*- single-visit pollination efficiency index.

**Fig 3:**
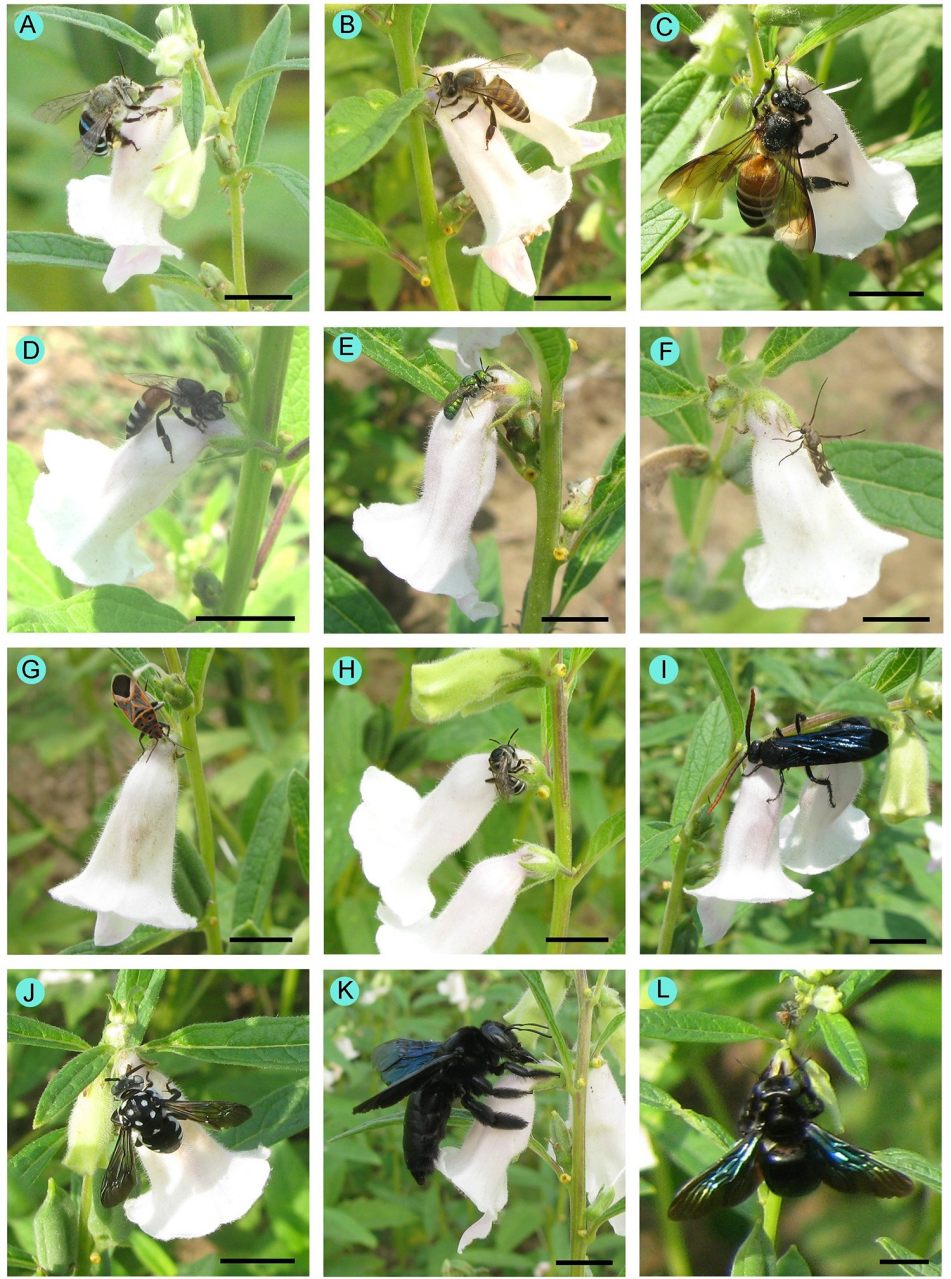
Some visitors robbing on sesame flowers. (A) *Amegilla zonata*, (B) *Apis cerana*, (C) *Apis dorsata*, (D) *Apis florea*, (E) *Ceratina binghami*, (F) *Eretmocera impactella*, (G) *Graptostethus servus*, (H) *Halictus acrocephalus*, (I) *Scolia soror*, (J) *Thyreus nitidulus*, (K) *Xylocopa fenestrata*, and (L) *Xylocopa latipes*. Scale bars = 10 mm.

We observed robbing visits for all the flower-visiting species except *Tetragonula iridipennis* (Table 1, Fig 3). The members of Hymenoptera (excluding *Ceratina* spp. and *Megachile monticola*) were primary robbers. Meanwhile, members of Hemiptera and Lepidoptera were secondary robbers. Some visitors (e.g., *Chalybion bengalense*, *Polistes tenebricosus*, *Utetheisa pulchella*, *Xylocopa aestuans*, *Xylocopa fenestrata* and *Xylocopa latipes*) were visited only as nectar-robbers. Black hairy flower wasps (*Scolia soror*) mostly visited sesame flowers as nectar robbers (92.75 ± 6.79% of robbing visits). Among the facultative robbers, higher percentages of robbing visits were recorded for *Apis cerana*, *Apis dorsata*, *Apis florea*, *Halictus acrocephalus* and *Pseudapis oxybeloides*. Nectar-robbing activity varied according to flowering periods (see S1 Table) and daytimes (see S2 Table). Percentages of robbing visits were higher in late flowering period (29.25 ± 16.07, 33.17 ± 17.92, 31.92 ± 19.02, 21.33 ± 13.74 for *Apis cerana*, *Apis dorsata*, *Apis florea*, *Ceratina binghami* and *Halictus acrocephalus*, respectively) than the early (8.17 ± 7.07, 9.33 ± 7.33, 9.17 ± 8.74, 7.75 ± 8.36, 8.92 ± 8.44 for *Apis cerana*, *Apis dorsata*, *Apis florea*, *Ceratina binghami* and *Halictus acrocephalus*, respectively) and mid flowering times (S1 Table). Daytime-wise robbing visits were higher in the late afternoon than in the early morning (S2 Table).

Flower visitation rate was higher in carpenter bees (*Xylocopa aestuans*, *Xylocopa amethystina*, *Xylocopa fenestrata* and *Xylocopa latipes*), and blue-banded bees (*Amegilla zonata*) (Table 1). The flower visitation rate significantly varied between non-robbing and robbing types of visitation (see S3 Table). The foraging rate was comparatively higher in the case of robbing visits than non-robbing visits (e.g., *Amegilla zonata*: non-robbing type: 13.72 ± 3.00 flowers/min, robbing type: 15.49 ± 3.43 flowers/min; *Apis dorsata*: non-robbing type: 6.22 ± 1.86 flowers/min, robbing type: 10.60 ± 1.99 flowers/min; *Ceratina binghami*: non-robbing type: 2.53 ± 1.04 flowers/min, robbing type: 5.24 ± 1.28 flowers/min; *Halictus acrocephalus*: non-robbing type: 2.97 ± 0.95 flowers/min, robbing type: 5.68 ± 1.35 flowers/min). Daytime-wise flower visitation rate significantly varied in some insect species like *Amegilla zonata*, *Apis cerana*, *Apis dorsata*, *Apis florea*, *Xylocopa aestuans*, *Xylocopa amethystina*, *Xylocopa fenestrata*, etc. (see S3 Table). However, in some insect species, the visitation rate does not vary according to daytime (e.g., *Ceratina binghami* and *Halictus acrocephalus*). We recorded a higher visitation rate for most insect species at 10.00–14.00 h (S3 Table). The amount of time spent per flower on a visit (i.e., flower handling time) was comparatively lower for carpenter bees (*Xylocopa aestuans*, *Xylocopa amethystina*, *Xylocopa fenestrata* and *Xylocopa latipes*) and blue-banded bees (*Amegilla zonata*), and higher for *Ceratina binghami* and *Halictus acrocephalus* (Table 1). The flower handling times for the visitors significantly varied between the non-robbing and robbing types of visitation (see S4 Table). They spent less time in robbing visits than non-robbing visits (*Amegilla zonata*: non-robbing: 3.01 ± 1.01 sec, robbing: 2.32 ± 0.61 sec; *Apis cerana*: non-robbing: 6.40 ± 2.76 sec, robbing: 3.52 ± 1.10 sec; *Apis dorsata*: non-robbing: 6.12 ± 2.66 sec, robbing: 3.78 ± 1.13 sec; *Apis florea*: non-robbing: 6.94 ± 2.37 sec, robbing: 3.61 ± 1.19 sec; *Ceratina binghami*: non-robbing: 17.29 ± 5.65 sec, robbing: 5.36 ± 1.28 sec; *Halictus acrocephalus*: non-robbing: 33.88 ± 12.35 sec, robbing: 3.61 ± 1.38 sec) (S4 Table).

The robbers visit flowers in a variable number of successive (i.e., in an uninterrupted manner) robbing visits. For honeybees, we recorded higher percentages for the group with the higher number of continuous robbing visits (i.e., > 30 successive visits) (Table 2). While in the case of solitary bees (e.g., *Amegilla zonata*, *Ceratina binghami*, *Halictus acrocephalus* and *Xylocopa amethystina*), greater proportions fall within the class ‘< 10 successive robbing visits’.

**Table 2:** Group-wise percentages of robbing visits for some dominant floral visitors of sesame.

Floral visitors	Number of successive robbing visits			
	< 10	10–20	>20–30	>30
*Amegilla zonata*	37.67 ± 29.10	21.33 ± 10.25	16.67 ± 8.64	24.33 ± 18.04
*Apis cerana*	24.33 ± 27.70	11 ± 3.74	11 ± 1.10	53.67 ± 29.46
*Apis dorsata*	34.33 ± 36.80	9.67 ± 3.44	10 ± 5.80	46 ± 32.72
*Apis florea*	7.67 ± 4.63	4 ± 2.83	3.67 ± 1.97	84.67 ± 8.26
*Ceratina binghami*	78.67 ± 11.50	13.33 ± 5.89	6 ± 4.38	2 ± 2.19
*Halictus acrocephalus*	53 ± 30.38	13.67 ± 3.88	9.33 ± 5.47	24 ± 23.08
*Xylocopa amethystina*	97.33 ± 2.42	2.67 ± 2.42	0	0

Values (%) are given as mean ± standard deviation.

### Drivers of robbing activity

#### Visitor’s body size and robbing activity

**Fig 4:**
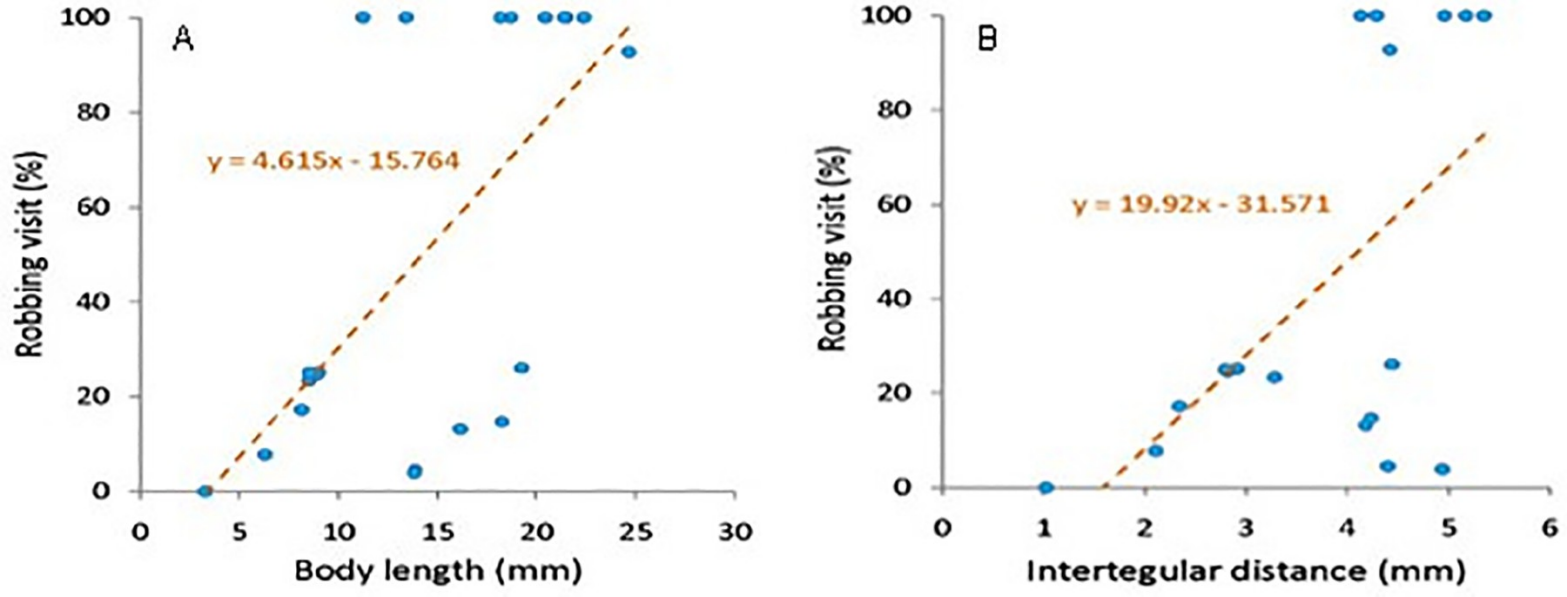
Scatter plots showing relationship between visitor’s body sizes and robbing activity (%). (A) body length vs. robbing activity, (B) intertegular distance vs. robbing activity of floral visitors.

The floral visitors of sesame were of different body sizes─ very small sized (e.g., *Tetragonula iridipennis*) to large sized (e.g., *Xylocopa latipes*). The body length ranged from 3.26 mm to 24.65 mm, and the intertegular distance ranged from 1.02 mm to 5.34 mm. The percentage of robbing visits significantly correlated with the visitors’ body size (body length vs. robbing: correlation coefficient ρ = 0.60, *p* < 0.01; intertegular distance vs. robbing: correlation coefficient ρ = 0.50, *p* < 0.05). Due to the narrow corollae tubes of sesame flowers, much larger bees (*Xylocopa aestuans*, *Xylocopa fenestrata*, and *Xylocopa latipes*) obligatorily visited the flowers as nectar robbers. However, some small and medium bees (which can enter the corolla tube easily) also showed robbing activity (Fig 4). Therefore, in addition to the body size of visitors, several other attributes (e.g., floral architecture and foraging behaviour of visitors) may also trigger robbing activity.

### Corolla tube length and robbing activity

The abundance of floral visitors significantly varied among the three corolla types, i.e., normal corolla, short-tube corolla without landing space, and short-tube corolla with landing space (*F*_2, 297_ = 24.67, *p* < 0.001). However, visitor’s abundance in a short-tube corolla with landing space (2.06 ± 1.29 visitors/flower/10 min) was almost similar to the normal flowers (2.24 ± 1.45 visitors/flower/10 min). In the case of a short-tube corolla without landing space, visitors faced difficulties to enter into the corolla tube. We observed that bees often attempted to visit the flowers but remained unsuccessful in these short-tube flowers; the visitor’s abundance was the lowest (1.08 ± 0.98 visitors/flower/10 min) among the three types (Table 3). Visitors showed both non-robbing and robbing visits in these two types of short-tube corolla (S2 Fig). However, the percentages of robbing visits differed among these three treatments (*F*_2, 27_ = 4.02, *p* < 0.05). The percentages of robbing visits were slightly lower in short-tube corolla types (11.97 ± 4.69% and 12.37 ± 7.97% for the short-tube without landing space and short-tube with landing space, respectively) than the normal flowers.

**Table 3:** Impact of corolla tube length on abundance (number of visitors/flower/10 min) and robbing activity of visitors (data recorded at 9.00–10.00 h).

Treatments	Visitor’s abundance	Robbing visit (%)
i. Normal flowers (i.e., control set)	2.24 ± 1.45	19.06 ± 5.72
ii. Flowers with short corolla tube without landing space	1.08 ± 0.98	12.37 ± 7.97
iii. Flowers with short corolla tube with landing space	2.06 ± 1.29	11.97 ± 4.62
Statistical analysis	*F*_2, 297_ = 24.67, *p* < 0.001	*F*_2, 27_ = 4.02, *p* < 0.05

Values are given as mean ± standard deviation.

#### Scarred corolla tube and robbing activity

The abundance of visitors does not differ between normal (i.e., control) flowers and artificially scarred flowers (*df* = 198, t = 0.43, *p* = 0.67). In the morning (6.00–7.00 h), abundance was 1.17 ± 1.19 visitors/flower/10 min and 1.10 ± 1.11 visitors/flower/10 min for normal and scarred flowers, respectively. The percentage of robbing visits is also almost similar in these two types of flowers (*df* = 18, t = -0.22, *p* = 0.83). Normal flowers received robbing visits at about 5.36 ± 6.20%, and scarred flowers at about 5.98 ± 6.65%. However, we found that *Ceratina binghami* performed robbing visits primarily to the flowers with scarred corolla tubes.

#### Floral rewards and robbing activity

**Fig 5:**
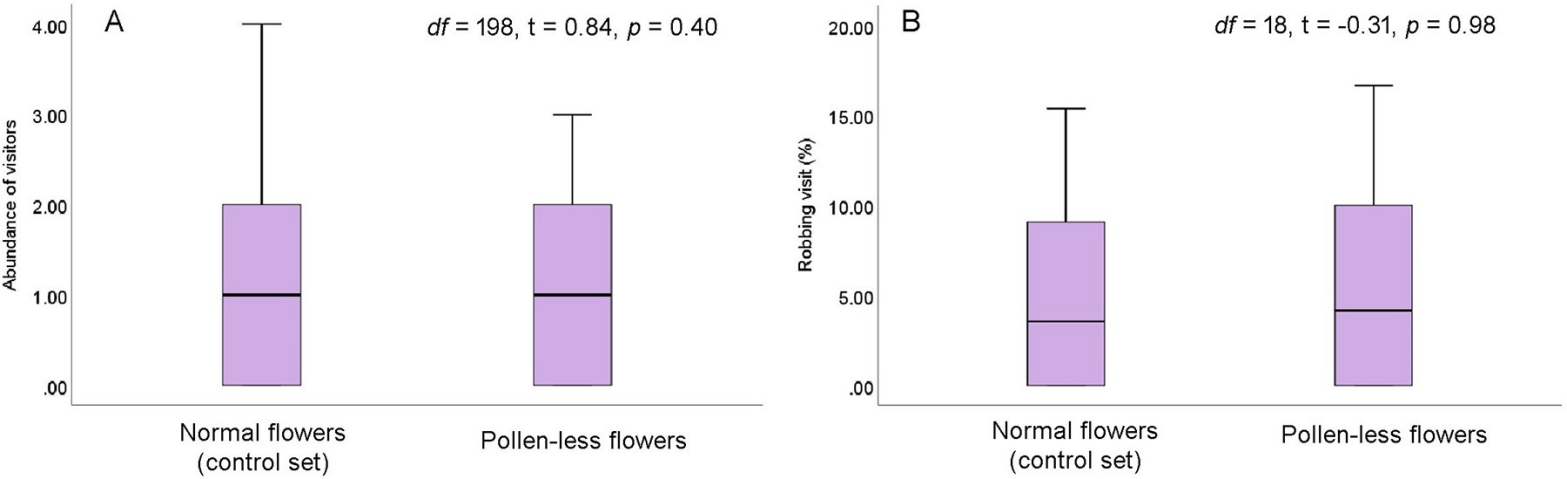
The abundance (number of visitors/flower/10 min) and robbing activity (%) of visitors on normal flowers (i.e., control set) and pollen-less flowers having nectar content (data recorded at 7.00–8.00 h). (A) abundance, (B) robbing visits of floral visitors.

**Fig 6:**
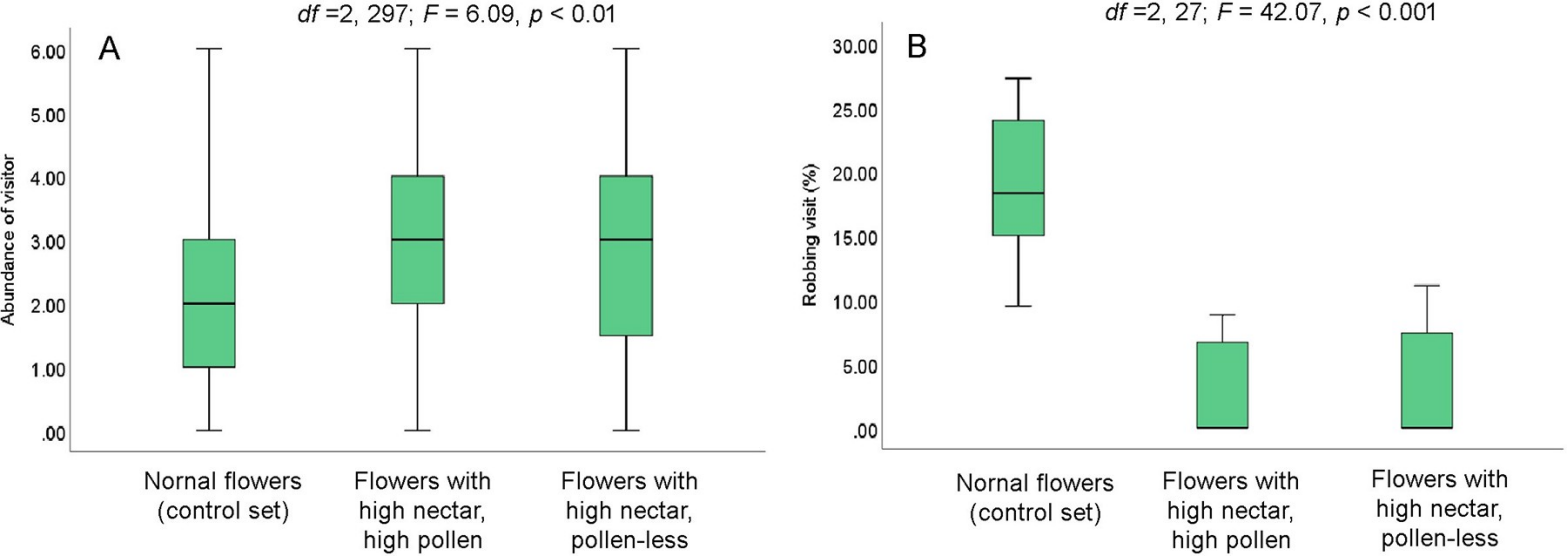
The abundance (number of visitors/flower/10 min) and robbing activity (%) of visitors on normal flowers (i.e., control set), flowers with high nectar and pollen content, and pollen-less flowers with nectar content (data recorded at 9.00–10.00 h). (A) abundance, (B) robbing visits of floral visitors.

Visitor’s abundance and percentage of robbing visits do not vary between the normal flowers (having a high amount of nectar and pollen in the early morning) and the pollen-less flowers (having a high amount of nectar in the early morning) (Fig 5). The visitor’s abundance was 1.17 ± 1.19 visitors/flower/10 min and 1.04 ± 0.98 visitors/flower/10 min for control (high nectar and pollen) and pollen less (with high nectar) flowers, respectively. The control flowers received about 5.36 ± 6.20% robbing visits, and pollen-less flowers received about 5.44 ± 6.18%. However, visitation patterns significantly differed between the normal flowers (comparatively with low resource availability in 9.00–10.00 h), flowers with higher nectar and pollen content, and pollen-less flowers with higher nectar content (Fig 6). The abundance of visitors was higher in flowers with higher resource content (3.01 ± 1.65 visitors/flower/10 min) than in the control flowers with lower resource content (2.24 ± 1.45 visitors/flower/10 min). Flowers with higher nectar content (both types─ pollen less and high pollen content flowers) predominantly received non-robbing visits (Fig 5), and percentages of robbing visits were very low (high nectar and high pollen: 2.26 ± 3.68%; high nectar and pollen less: 2.99 ± 4.24%). In contrast, control flowers with little resources at that time received a significant percentage of robbing visits (19.06 ± 5.72%).

#### Resource collecting task allocation and robbing activity

Within the two categories (i.e., robbers and non-robbers), specialized nectar foragers and mixed foragers were found. The percentages of specialized nectar foragers, as well as mixed foragers, were significantly varied between the non-robber and robber categories (see S5 Table). The percentages of specialized nectar foragers were comparatively higher in robber category (*Apis cerana*: 75.50 ± 5.99%; *Apis dorsata*: 78 ± 9.19%; *Apis florea*: 76.50 ± 5.80%) than in the non-robber category (*Apis cerana*: 59 ± 6.58%; *Apis dorsata*: 60.50 ± 8.32%; *Apis florea*: 58.50 ± 6.69%). In contrast, percentages of mixed foragers were lower in robbers (*Apis cerana*: 24.50 ± 5.99%; *Apis dorsata*: 22 ± 9.19%; *Apis florea*: 23.50 ± 5.80%) than in the non-robbers (*Apis cerana*: 41 ± 6.58%; *Apis dorsata*: 39.50 ± 8.32%; *Apis florea*: 41.50 ± 6.69%).

### Impact of legitimate and robbing visits on plant reproduction

Sesame is autogamous, resulting in fruit and seed sets in pollinator-exclusion treatment. However, fruit and seed sets significantly differed between open and pollinator-exclusion treatments (see Table 4). Fruit and seed sets remain lower in bagged flowers (fruit set: 83 ± 9.49%; seed set: 43.17 ± 20.44 per flower) than in open field condition (fruit set: 96 ± 6.99%; seed set: 54.07 ± 15.28 per flower). The seed set resulted from a single legitimate visit, and a single illegitimate visit had no significant difference (*Amegilla zonata*: *df* = 45, t = 0.71, *p* = 0.48; *Apis cerana*: *df* = 73, t = 1.03, *p* = 0.30; *Apis dorsata*: *df* = 63, t = 0.96, *p* = 0.34). Fruit and seed sets resulted from the three types of multiple visits [(i) multiple legitimate visits; (ii) initial legitimate visit, then followed by many mixed visits, and (iii) initial robbing visit, then followed by multiple mixed visits] did not significantly differ (see Table 4). Like that of open field condition, three types of multiple visits resulted in almost similar fruit set (multiple legitimate visits: 94.67 ± 8.64%; initial legitimate visit: 93 ± 9.09%; initial illegitimate visit: 92.50 ± 16.87%) and seed set (multiple legitimate visits: 52.76 ± 14.47 seeds per flower; initial legitimate visit: 51.85 ± 15.88 seeds per flower; initial illegitimate visit: 50.70 ± 14.48 seeds per flower).

**Table 4:** Fruit and seed sets of sesame in different pollination systems.

Pollination systems	Fruit set (%)	Number of Seeds per flower	Statistical analysis
Open pollination	96 ± 6.99	54.07 ± 15.28	Fruit set: *df* = 18, t = 3.49, *p* < 0.01 Seed set: *df* = 58, t = 2.34, *p* < 0.05
Pollinator-exclusion	83 ± 9.49	43.17 ± 20.44	
Single legitimate visit			
• *Amegilla zonata*	89.33 ± 9.83	48.41 ± 14.84	
• *Apis cerana*	90.55 ± 0.50	48.55 ± 16.49	
• *Apis dorsata*	91.28 ± 8.65	49.48 ± 16.39	
• *Apis florea*	90.29 ± 13.64	46.40 ± 18.20	
• *Ceratina binghami*	86.28 ± 8.18	46.16 ± 18.23	
• *Halictus acrocephalus*	90.17 ± 9.36	47.97 ± 16.88	
Single illegitimate visit			
• *Amegilla zonata*	86.67 ± 12.64	44.80 ± 19.82	
• *Apis cerana*	87 ± 12.04	44.09 ± 18.23	
• *Apis dorsata*	87 ± 12.04	45.10 ± 19.04	
Multiple visits			
• Multiple legitimate visits	94.67 ± 8.64	52.76 ± 14.47	Fruit set: GLM, Wald *χ*^*2*^ = 0.14, *df* = 1, *p* = 0.71 Seed set: GLM, Wald *χ*^*2*^ = 2.10, *df* = 1, *p* = 0.15
• Initial legitimate visit, followed by multiple mixed type visits	93 ± 9.09	51.85 ± 15.88	
• Initial illlegitimate visit, followed by multiple mixed type visits	92.50 ± 16.87	50.70 ± 14.48	

Values are given as mean ± standard deviation.

## Discussion

Several insect species visited sesame flowers, with the dominance of hymenopterans members, including honeybees, halictidae and carpenter bees. These findings are in close agreement with other studies, such as Viraktmath et al. [25] and Mahfouz et al. [26]. Most visitors showed both legitimate and illegitimate (here, nectar robbing) types of visitation. This is consistent with the existing literature; many studies have demonstrated that Hymenoptera (especially bees) are the most common robbers and use mixed foraging strategies [3,13,27]. Larger carpenter bees (some wasps also) visited as obligatorily nectar robbers. In contrast, honeybees and other small to medium size bees acted as facultative nectar robbers. There are two standpoints in explaining why insects forage as nectar robbers. One is that insects can only illegitimately get food because of the mismatch of the morphologies of insects and floral parts. The other point of view argues that nectar robbing is relatively more efficient, thus, a more energy-saving way for insects to get nectar from flowers [28].

The floral visitors maintained both non-robbing (= legitimate) and robbing visits to a robbed flower. By robbing visits, flowers were not mutilated badly; therefore, visitors also carried legitimate visits to the robbed flowers. Two hypotheses can explain this phenomenon. Firstly, the pollinators do not distinguish between robbed and unrob flowers and keep their legitimate visits on robbed flowers [29,30]. Secondly, the pollinators distinguish robbed flowers from unrob flowers and visit robbed flowers as secondary nectar robbers [31–33].

In the case of robbing visits, they spent less time on a flower, and the visitation rate was comparatively higher than non-robbing legitimate visits. The positive effect of robbing on the foraging efficiency of visitors is well established in different plant species [34,35], including a relative wild of sesame, i.e., *Sesamum radiatum* [20]. By robbing, they can reduce the foraging cost (energy consumption) concerning the collection of floral resources.

The proportion of robbing visits remains higher during the late flowering period, about 10.00–14.00 h. Time period-wise differences in robbing were also documented for other plant species [27]. In the late flowering period, nectar yield may lower, and visitors face difficulty accessing the nectar, so they intend to rob the flowers more frequently. Additionally, in the late flowering period, a lower number of sesame fields, a smaller number of flowers and a higher abundance of insect visitors may result in higher competition among the floral visitors. For that, visitors cherished collecting floral rewards (mainly nectar in sesame) in a quick manner by robbing visits. As nectar-robbing behaviour is driven by competition for resources, and can learn it from each other [27,36].

Visitor’s body size and robbing visits were significantly correlated. Larger carpenter bees (*Xylocopa aestuans*, *Xylocopa fenestrata* and *Xylocopa latipes*) obligatorily robbed sesame flowers because they cannot able to enter into the narrow corolla tube of flowers. Besides the large-sized insects, small-sized bees also visited sesame flowers as robbers. Therefore, a visitor’s body size may be treated as one of the determinants that govern robbing visits, but not only a crucial factor influencing the robbing behaviour of bees. Valdivia et al. [37] also found a significant relationship between a bee’s body size and nectar robbing. However, they stated that smaller bees robbed more frequently, which was the reverse of our findings; it solely depends on flower morphologies and foraging strategies of visitors. Stanley and Cosnett [27] stated that bumble bee body size and nectar-robbing behaviour do not have a significant relationship. Besides body size, tongue length may limit legitimate visits [27,38]. But, we did not consider this parameter in our present study.

The corolla tube length is another floral trait that influences robbing behaviour. In our experiment, flowers having shorter corollae tubes received fewer robbing visits than in longer (i.e., normal flowers) types. This aligns with existing literature demonstrating that plants with long flowers are more likely to be robbed [4,16,39]. In flowers with long-tube corollae, visitors need more time and energy to collect the nectar than in flowers with short-tube corollae. However, in our experiment, the artificially shortening of the corolla tube may mutilate the flowers. It is unlikely that the shortening of the tube alone is a factor, but the damage is also. Many smaller flowers with short corolla tubes, like *Leucas aspera* [40], have also received robbing visits.

Sesame flowers received many robbing visits. As a result of repeated piercing, a small hole (or scar) was formed at the base of the corolla tube. The scar did not influence the subsequent visitation pattern. However, small bees (*Ceratina* spp.) performed robbing mostly on flowers with scars on corolla tubes. It may be due to their secondary and facultative type of nectar robbers. They only steal nectar from the robbed flowers, having a unique pathway (i.e., a scar at the base of the corolla tube). Leadbeater and Chittka [36] discovered that learning nectar robbing by one individual facilitates its adoption by others.

Flowers with a higher amount of nectar content received the least robbing visits. In comparison, flowers with a lesser amount of nectar perceived significant robbing visits. That means the robbing behaviour of bees largely depends on the availability and accessibility of nectar. The dependency of robbing on the accessibility constraints and nectar volume was established by several workers [19,41,42]. In contrast, the pollen content of sesame flowers does not significantly affect robbing activity. This may be due to the less preference for sesame pollen by most visitors.

Floral resource collection task allocation (i.e., specialized nectar and pollen foragers and mixed foragers) influences the robbing activity of bees. The results of higher percentages of nectar foragers in the robber’s category indicate that the specialized nectar foragers were more intent on stealing flowers than mixed foragers. Unlike mixed foragers, specialized nectar foragers do not collect pollen; their goal is to collect nectar easily and quickly. Therefore, they choose to rob flowers more frequently than mixed foragers.

Nectar robbing may have positive, negative or neutral effects on plant reproduction [13]. The direction of the impact of robbing on plant reproductive fitness is also predicted by the mating system of plants [32,43]. It is believed that self-incompatible plants will suffer in terms of fruit and seed set, but self-compatible plants will have no detrimental influence on plant reproductive function. Robbing can negatively affect the plant in several ways, including damaging the reproductive structure while probing the flowers [44], aggressively interacting with pollinators [45], and making robbed flowers less attractive to pollinators [32,46]. However, in the case of autogamous and facultative outcrossing species, robbing has mostly either neutral or positive effects on plant reproduction [30,32,43]. In the case of positive impacts, nectar robbers’ bodies may come into contact with plant sex organs during their visit to the flowers, resulting in pollination. Alternately, during their robbing visits, pollen may be loaded to the stigma, probably by jerking of the corolla tube and close association of stigma and anthers; robbing can promote foraging and pollinator movement, thus increasing fruit and seed sets [47]. In sesame, robbing had no significant effect on plant reproduction. However, we did not collect data on flowers received exclusively multiple robbing visits; instead, we gathered fruit and seed sets data from single robbing visits and multiple mixed visits starting with a robbing visit. Nectar robbers have a neutral effect in that they ruin the corollae of flowers but do not touch the sex organs or destroy the ovules [28]. Furthermore, they did not make the flowers less attractive to pollinators, as flowers also received several visits after getting a robbing visit.

## Conclusions

Sesame flowers are visited by several insect species, primarily members of Hymenoptera. The most effective pollinators were *Amegilla zonata*, *Apis cerana*, *Apis dorsata*, *Apis florea*, *Ceratina binghami*, *Halictus* acrocephalus and *Xylocopa amethystina*. The nectar-robbing phenomenon is common for almost all visitors with variable percentages. For that, sesame can be used as a model plant for studying the nectar-robbing behaviour of bees. Primary robbers were the members of Hymenoptera (excluding *Ceratina* spp. and *Megachile monticola*). Nectar robbing behaviour is influenced by multiple attributes, including the visitor’s body size, the corolla tube length (longer tube length received greater robbing visits), the flowering period (in late flowering phase, percentages of robbing visits were higher than early and mid flowering phases), the daytime (in afternoon, percentages of robbing visits were higher than early morning), floral resource availability (flowers with higher nectar content received least robbing visits), and resource collecting task allocation of bees. Robbing visits depend on the amount of nectar and accessibility of nectar rather than the pollen content of flowers. In the case of robbing visits, flower handling time (time spent per flower) was lower, and the visitation rate was higher than in non-robbing visits. Regarding reproductive mode, sesame is auto-pollinated, while pollinator visits increased reproductive success. Individual flowers generally received multiple visits─ multiple legitimate visits or mixed types of visitation (legitimate and robbing visits). Initial robbing visits (followed by multiple mixed visits) do not affect the fruit and seed sets of the plant species. Therefore, robbing activity can help visitors collect more nectar quickly but without impacting the reproduction of the plant species.

## Supporting information

S1 Fig Flowers of sesame. (A) normal flower, (B) short corolla tube flower without landing space, and (C) short-tube flower with landing space. Scale bars = 10 mm.
(JPG)

S2 Fig Legitimate visits on short-tube flowers (A) without landing space, (B) with landing space, and illegitimate (robbing) visit on short-tube flower with landing space. Scale bars = 10 mm.
(JPG)

S1 Table Robbing visits (%) of dominant visitors of sesame across flowering period. (DOCX)

S2 Table Daytime wise robbing visits (%) of floral visitors on sesame flowers. (DOCX)

S3 Table Daytime wise flower visitation rate of visitors on sesame flowers. (DOCX)

S4 Table Visitation patterns (robbing and non-robbing) wise flower handling time (amount of time spent on a flower per visit) of visitors on sesame flowers. (DOCX)

S5 Table Percentages of specialized nectar and mixed foragers were recorded for non-robbers and robbers. (DOCX)
