# Supplementary figures and images for: Floral visitors of sesame (Sesamum indicum L.): Elucidating their nectar-robbing behaviour and impacts on the plant reproduction
Source: PLoS One. 2024 Apr 18;19(4):e0300398. doi: 10.1371/journal.pone.0300398 (PMC11025750; doi:10.1371/journal.pone.0300398)

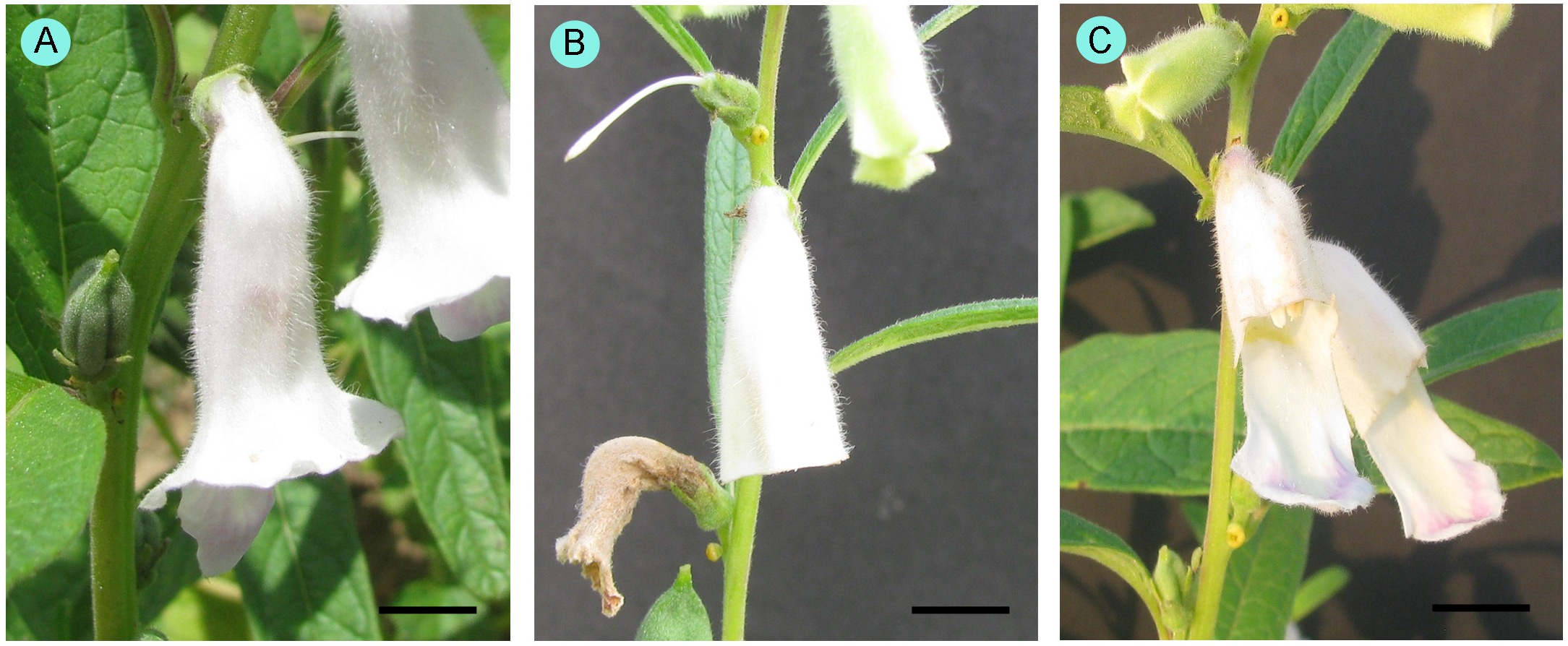

Supplement: S1 Fig — (A) normal flower, (B) short corolla tube flower without landing space, and (C) short-tube flower with landing space. Scale bars = 10 mm. (JPG) [file pone.0300398.s001.jpg]

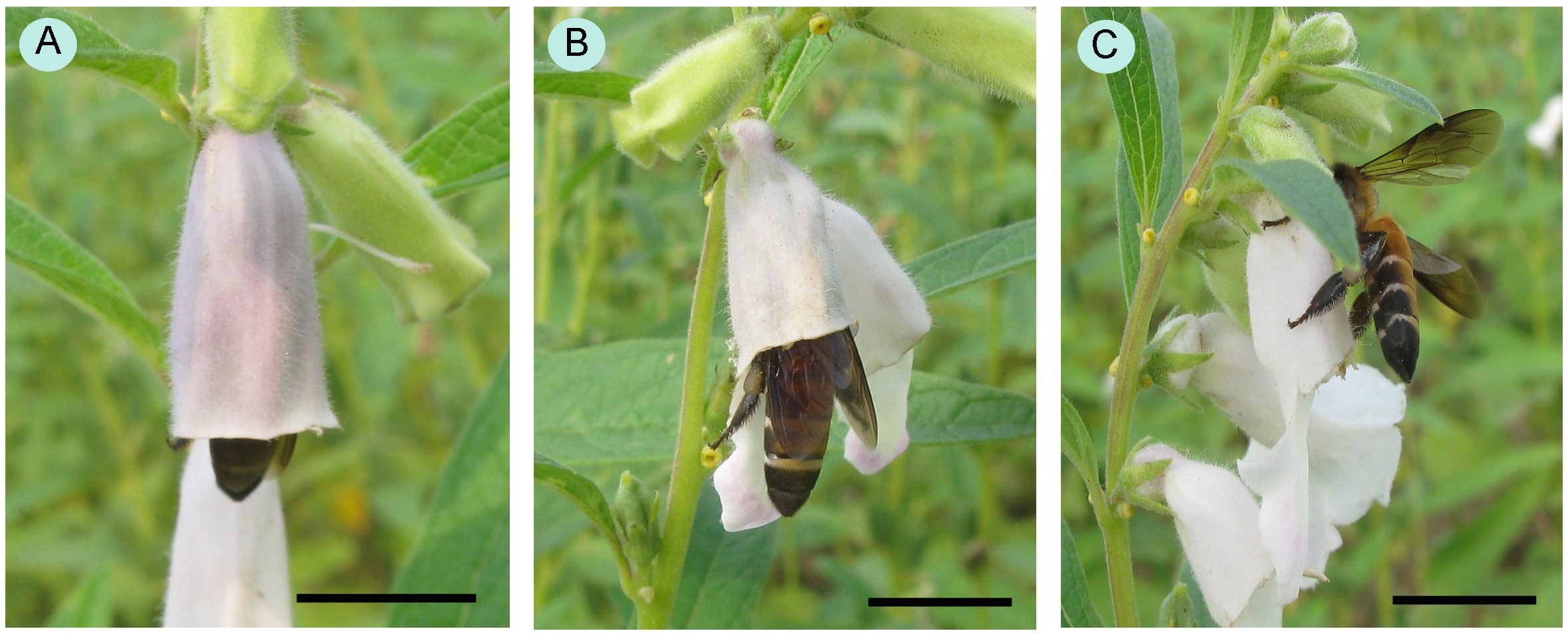

Supplement: S2 Fig — Legitimate visits on short-tube flowers (A) without landing space, (B) with landing space, and illegitimate (robbing) visit on short-tube flower with landing space. Scale bars = 10 mm. (JPG) [file pone.0300398.s002.jpg]
