# Supplementary material for: Floral visitors of sesame (Sesamum indicum L.): Elucidating their nectar-robbing behaviour and impacts on the plant reproduction
Source: PLoS One. 2024 Apr 18;19(4):e0300398. doi: 10.1371/journal.pone.0300398 (PMC11025750; doi:10.1371/journal.pone.0300398)
Supplement: S1 Table — (DOCX) [file pone.0300398.s003.docx]

**S1 Table.** Flowering period wise robbing visits (%) of some dominant visitors of sesame.

| Floral visitors | Flowering period wise robbing visit (%) | | | Statistical analysis |
| --- | --- | --- | --- | --- |
|  | Early | Middle | Late |  |
| *Apis cerana* | 8.17^c^ ± 7.07 | 23.43^b^ ± 14.45 | 29.25^a^ ± 16.07 | *F*_2, 177_ = 41.25, *p* < 0.001 |
| *Apis dorsata* | 9.33^c^ ± 7.33 | 26.08^b^ ± 15.52 | 33.17^a^ ± 17.92 | *F*_2, 177_ = 43.79, *p* < 0.001 |
| *Apis florea* | 9.17^c^ ± 8.74 | 25.17^b^ ± 16.34 | 31.92^a^ ± 19.02 | *F*_2, 177_ = 34.84, *p* < 0.001 |
| *Ceratina binghami* | 7.75^c^ ± 8.36 | 17.33^b^ ± 12.84 | 21.33^a^ ± 13.74 | *F*_2, 177_ = 20.71, *p* < 0.001 |
| *Halictus acrocephalus* | 8.92^c^ ± 8.44 | 24.58^b^ ± 16.11 | 31.17^a^ ± 18.76 | *F*_2, 177_ = 34.44, *p* < 0.001 |

Values are given in mean ± standard deviation. Row-wise values followed by the different superscript letter are significantly differed at p < 0.05, DMRT test.
