# Supplementary material for: Floral visitors of sesame (Sesamum indicum L.): Elucidating their nectar-robbing behaviour and impacts on the plant reproduction
Source: PLoS One. 2024 Apr 18;19(4):e0300398. doi: 10.1371/journal.pone.0300398 (PMC11025750; doi:10.1371/journal.pone.0300398)
Supplement: S2 Table — (DOCX) [file pone.0300398.s004.docx]

**S2 Table.** Daytime wise robbing visits (%) of floral visitors on sesame flowers.

| Floral visitors | Daytime (h) wise robbing visits (%) | | | | | | Statistical analysis |
| --- | --- | --- | --- | --- | --- | --- | --- |
|  | 6.00–8.00 | 8.00–10.00 | 10.00–12.00 | 12.00–14.00 | 14.00–16.00 | 16.00–18.00 |  |
| Hemiptera |  |  |  |  |  |  |  |
| *Graptostethus servus* | 100 | 100 | 100 | 100 | 100 | 100 |  |
| Hymenoptera |  |  |  |  |  |  |  |
| *Amegilla zonata* | 2 ± 2.58 | 9 ± 6.58 | 15 ± 9.72 | 17.50 ± 9.79 | 19.50 ± 9.26 | 16.50 ± 8.18 | *F*_5, 54_ = 6.58, *p* < 0.001 |
| *Apis cerana* | 5.50 ± 5.50 | 11 ± 8.10 | 23.50 ± 9.44 | 33 ± 10.33 | 35 ± 10 | 32.50 ± 10.34 | *F*_5, 54_ = 18.86, *p* < 0.001 |
| *Apis dorsata* | 7 ± 5.87 | 14.50 ± 8.96 | 27 ± 11.11 | 35.50 ± 13.01 | 38.50 ± 11.56 | 34 ± 11.97 | *F*_5, 54_ = 14.11, *p* < 0.001 |
| *Apis florea* | 4 ± 3.94 | 10 ± 8.82 | 24 ± 9.37 | 36.50 ± 9.73 | 41.50 ± 9.44 | 35 ± 9.13 | *F*_5, 54_ = 31.35, *p* < 0.001 |
| *Ceratina binghami* | 1.50 ± 2.42 | 8 ± 7.15 | 17 ± 10.85 | 25 ± 11.06 | 28 ± 7.53 | 24.50 ± 10.12 | *F*_5, 54_ = 3.99, *p* < 0.001 |
| *Ceratina compacta* | 0 | 3.50 ± 4.12 | 9 ± 6.58 | 11.50 ± 4.74 | 13.50 ± 4.74 | 9.50 ± 3.69 | *F*_5, 54_ = 13.10, *p* < 0.001 |
| *Chalybion bengalense* | 100 | 100 | 100 | 100 | 100 | - |  |
| *Halictus acrocephalus* | 3.50 ± 4.12 | 9.50 ± 6.43 | 21 ± 8.10 | 36.50 ± 10.01 | 38 ± 7.53 | 39 ± 8.43 | *F*_5, 54_ = 41.42, *p* < 0.001 |
| *Megachile monticola* | 1.50 ± 3.37 | 4 ± 5.16 | 5.50 ± 4.97 | 7 ± 5.37 | 6.50 ± 4.74 | 3 ± 4.22 | *F*_5, 54_ = 11.43, *p* < 0.001 |
| *Polistes tenebricosus* | 100 | 100 | 100 | 100 | - |  |  |
| *Pseudapis oxybeloides* | 3 ± 3.50 | 10 ± 4.71 | 22.50 ± 9.20 | 38 ± 13.37 | 41 ± 13.50 | 36 ± 10.75 | *F*_5, 54_ = 25.40, *p* < 0.001 |
| *Scolia soror* | 88 ± 8.56 | 89.50 ± 6.85 | 92 ± 7.15 | 94.50 ± 5.50 | 96 ± 3.94 | 96.50 ± 4.12 | *F*_5, 54_ = 3.15, *p* < 0.05 |
| *Tetragonula iridipennis* | 100 | 100 | 100 | 100 | 100 | - |  |
| *Thyreus nitidulus* | 4 ± 3.94 | 9.50 ± 6.43 | 13.50 ± 8.18 | 18 ± 7.15 | 22 ± 8.23 | 21.50 ± 7.47 | *F*_5, 54_ = 10.18, *p* < 0.001 |
| *Xylocopa aestuans* | 100 | 100 | 100 | 100 | 100 | - |  |
| *Xylocopa amethystina* | 0 | 3 ± 3.50 | 4.50 ± 3.69 | 6.50 ± 4.74 | 7 ± 4.83 | 2.50 ± 3.54 | *F*_5, 54_ = 4.95, *p* < 0.001 |
| *Xylocopa fenestrata* | 100 | 100 | 100 | 100 | 100 | 100 |  |
| *Xylocopa latipes* | 100 | 100 | 100 | 100 | 100 | 100 |  |
| Lepidoptera |  |  |  |  |  |  |  |
| *Eretmocera impactella* | 100 | 100 | 100 | 100 | 100 | 100 |  |
| *Utetheisa pulchella* | 100 | 100 | 100 | 100 |  |  |  |

Values are given in mean ± standard deviation.
