# Supplementary material for: Floral visitors of sesame (Sesamum indicum L.): Elucidating their nectar-robbing behaviour and impacts on the plant reproduction
Source: PLoS One. 2024 Apr 18;19(4):e0300398. doi: 10.1371/journal.pone.0300398 (PMC11025750; doi:10.1371/journal.pone.0300398)
Supplement: S3 Table — (DOCX) [file pone.0300398.s005.docx]

**S3 Table.** Daytime wise flower visitation rate of visitors on sesame flowers.

| Floral visitors | VP | Daytime (h) | | | | | | Statistical analysis | |
| --- | --- | --- | --- | --- | --- | --- | --- | --- | --- |
|  |  | 6.00–8.00 | 8.00–10.00 | 10.00–12.00 | 12.00–14.00 | 14.00–16.00 | 16.00–18.00 | NR vs. R  (*df* = 238) | Considering daytime (*df* = 5, 114) |
| Hemiptera |  |  |  |  |  |  |  |  |  |
| *Graptostethus servus* | R | - | - | - | - | - | - |  |  |
| Hymenoptera |  |  |  |  |  |  |  |  |  |
| *Amegilla zonata* | NR | 11.80 ± 2.31 | 13.70 ± 2.98 | 15.10 ± 2.88 | 15.05 ± 3.10 | 14.15 ± 3.08 | 12.50 ± 2.28 | t = -4.27, *p* < 0.001 | *F* = 4.63, *p* < 0.001 |
|  | R | 13.25 ± 2.55 | 15.15 ± 2.85 | 17.40 ± 3.90 | 17.75 ± 3.13 | 16.10 ± 2.73 | 13.30 ± 2.56 |  | *F* = 8.51, *p* < 0.001 |
| *Apis cerana* | NR | 3.95 ± 0.89 | 6 ± 2 | 6.65 ± 1.57 | 7.05 ± 1.47 | 6.30 ± 1.59 | 5.05 ± 1.54 | t = -19.71, *p* < 0.001 | *F*= 11.00, *p* < 0.001 |
|  | R | 9.90 ± 1.89 | 11.15 ± 2.01 | 12.05 ± 2.19 | 11.65 ± 2.25 | 10.60 ± 2.28 | 10.10 ± 1.45 |  | *F*= 3.56, *p* < 0.01 |
| *Apis dorsata* | NR | 4.50 ± 0.69 | 6.35 ± 1.18 | 7.20 ± 2.02 | 7.40 ± 2.06 | 6.60 ± 1.70 | 5.30 ± 1.42 | t = -17.57, *p* < 0.001 | *F*= 10.05, *p* < 0.001 |
|  | R | 9.50 ± 1.85 | 10.55 ± 1.85 | 11.30 ± 1.95 | 11.55 ± 1.99 | 11 ± 2.03 | 9.70 ± 1.56 |  | *F*= 4.06, *p* < 0.01 |
| *Apis florea* | NR | 3.40 ± 0.99 | 5.15 ± 1.73 | 5.55 ± 1.82 | 5.90 ± 1.86 | 5.20 ± 1.64 | 4.50 ± 1.28 | t = -18.16, *p* < 0.001 | *F*= 6.32, *p* < 0.001 |
|  | R | 8.80 ± 1.79 | 9.25 ± 1.94 | 9.55 ± 1.99 | 9.40 ± 1.85 | 9.15 ± 1.87 | 8.85 ± 1.73 |  | *F*= 0.51, *p* = 0.77 |
| *Ceratina binghami* | NR | 2.20 ± 0.95 | 2.45 ± 1.10 | 2.55 ± 1.05 | 2.90 ± 1.07 | 2.65 ± 1.09 | 2.45 ± 1.00 | t = -17.93, *p* < 0.001 | *F*= 1.00, *p* = 0.42 |
|  | R | 4.80 ± 1.01 | 5.10 ± 1.12 | 5.30 ± 1.26 | 5.60 ± 1.47 | 5.50 ± 1.40 | 5.15 ± 1.39 |  | *F*= 1.03, *p* = 0.42 |
| *Ceratina compacta* | NR | 1.90 ± 0.79 | 2.10 ± 1.02 | 2.20 ± 1.01 | 2.35 ± 0.93 | 2.20 ± 0.89 | 2.10 ± 0.79 | t = -19.14, *p* < 0.001 | *F*= 0.54, *p* = 0.74 |
|  | R | 4.35 ± 0.88 | 4.60 ± 1.05 | 4.80 ± 1.15 | 4.85 ± 1.35 | 4.65 ± 1.09 | 4.40 ± 0.99 |  | *F*= 0.69, *p* = 0.63 |
| *Chalybion bengalense* | R | 2.75 ± 0.79 | 2.85 ± 0.88 | 2.90 ± 1.02 | 2.70 ± 0.86 | 2.45 ± 0.94 | - | - | - |
| *Halictus acrocephalus* | NR | 2.60 ± 0.99 | 2.90 ± 0.97 | 3.05 ± 1.05 | 3.30 ± 0.86 | 3.10 ± 0.91 | 2.85 ± 0.88 | t = -17.98, *p* < 0.001 | *F*= 1.29, *p* = 0.27 |
|  | R | 5.25 ± 0.91 | 5.55 ± 1.10 | 5.90 ± 1.33 | 6.20 ± 1.51 | 5.80 ± 1.47 | 5.40 ± 1.60 |  | *F*= 1.36, *p* = 0.24 |
| *Megachile monticola* | NR | 4.50 ± 1.05 | 6.15 ± 2.08 | 6.85 ± 1.63 | 7.40 ± 1.35 | 6.55 ± 1.61 | 5.50 ± 1.54 | t = -19.55, *p* < 0.001 | *F*= 8.64, *p* < 0.001 |
|  | R | 10.10 ± 2.00 | 11.30 ± 2.00 | 12.20 ± 2.17 | 11.95 ± 2.04 | 10.80 ± 2.12 | 10.30 ± 1.56 |  | *F*= 3.74, *p* < 0.01 |
| *Polistes tenebricosus* | R | 3.15 ± 0.59 | 3.25 ± 0.64 | 3.30 ± 0.80 | 3.20 ± 0.70 | - | - | - | - |
| *Pseudapis oxybeloides* | NR | 2.80 ± 1.06 | 3.20 ± 0.83 | 3.45 ± 1.00 | 3.65 ± 1.00 | 3.25 ± 0.97 | 2.95 ± 0.87 | t = -17.70, *p* < 0.001 | *F*= 2.13, *p* = 0.06 |
|  | R | 5.40 ± 0.94 | 5.85 ± 1.18 | 6.20 ± 1.32 | 6.45 ± 1.39 | 6.05 ± 1.43 | 5.50 ± 1.57 |  | *F*= 1.89, *p* = 0.10 |
| *Scolia soror* | R | 3.90 ± 1.12 | 4.15 ± 1.09 | 4.40 ± 1.10 | 4.50 ± 1.00 | 4 ± 1.03 | 3.85 ± 0.93 |  | *F*= 1.31, *p* = 0.26 |
| *Tetragonula iridipennis* | NR | 2.25 ± 0.85 | 2.55 ± 0.94 | 2.75 ± 1.07 | 2.95 ± 1.00 | 2.70 ± 0.98 | 2.40 ± 0.75 |  | *F*= 1.45, *p* = 0.21 |
| *Thyreus nitidulus* | NR | 4.25 ± 1.07 | 5.75 ± 1.80 | 6.10 ± 1.48 | 6.60 ± 1.50 | 6.10 ± 1.62 | 4.90 ± 1.37 | t = -20.69, *p* < 0.001 | *F*= 6.89, *p* < 0.001 |
|  | R | 9.65 ± 1.73 | 10.90 ± 1.89 | 11.75 ± 2.10 | 11.45 ± 2.06 | 10.40 ± 2.04 | 9.50 ± 1.57 |  | *F*= 4.71, *p* < 0.001 |
| *Xylocopa aestuans* | R | 11.30 ± 2.89 | 12.05 ± 3.17 | 12.95 ± 3.59 | 13.50 ± 3.62 | 12.25 ± 3.02 | 10.40 ± 2.68 |  | *F*= 2.46, *p* < 0.05 |
| *Xylocopa amethystina* | NR | 12.80 ± 2.76 | 14.60 ± 2.93 | 17.05 ± 3.87 | 17.20 ± 2.91 | 15.65 ± 2.72 | 12.95 ± 2.58 |  | *F*= 8.33, *p* < 0.001 |
| *Xylocopa fenestrata* | R | 14 ± 2.99 | 17.10 ± 4.42 | 19.40 ± 4.28 | 18.40 ± 2.96 | 18 ± 3.48 | 14.70 ± 2.83 |  | *F*= 8.33, *p* < 0.001 |
| *Xylocopa latipes* | R | 13.40 ± 2.70 | 16.30 ± 4.44 | 18.40 ± 4.48 | 17.75 ± 3.27 | 17.25 ± 3.37 | 14.25 ± 2.86 |  | *F*= 6.21, *p* < 0.001 |
| Lepidoptera |  |  |  |  |  |  |  |  |  |
| *Eretmocera impactella* | R | - | - | - | - | - | - |  |  |
| *Utetheisa pulchella* | R | - | - | - | - | - | - |  |  |

VP: visitation pattern, R: robbing, NR: non-robbing; values are given in mean ± standard deviation.
