# Supplementary material for: Floral visitors of sesame (Sesamum indicum L.): Elucidating their nectar-robbing behaviour and impacts on the plant reproduction
Source: PLoS One. 2024 Apr 18;19(4):e0300398. doi: 10.1371/journal.pone.0300398 (PMC11025750; doi:10.1371/journal.pone.0300398)
Supplement: S4 Table — (DOCX) [file pone.0300398.s006.docx]

**S4 Table.** Visitation patterns (robbing and non-robbing) wise flower handling time (amount of time spent on a flower per visit) of visitors on sesame flowers.

| Floral visitors | Flower handling time (second) | | |
| --- | --- | --- | --- |
|  | Non-robbing visit | Robbing visit | Statistical analysis |
| Hemiptera |  |  |  |
| *Graptostethus servus* | - | - | - |
| Hymenoptera |  |  |  |
| *Amegilla zonata* | 3.01 ± 1.01 | 2.32 ± 0.61 | *df* = 238, t = 6.34, p < 0.001 |
| *Apis cerana* | 6.40 ± 2.76 | 3.52 ± 1.10 | *df* = 238, t = 10.62, p < 0.001 |
| *Apis dorsata* | 6.12 ± 2.66 | 3.78 ± 1.13 | *df* = 238, t = 8.88, p < 0.001 |
| *Apis florea* | 6.94 ± 2.37 | 3.61 ± 1.19 | *df* = 238, t = 13.74, p < 0.001 |
| *Ceratina binghami* | 17.29 ± 5.65 | 5.36 ± 1.28 | *df* = 238, t = 22.55, p < 0.001 |
| *Ceratina compacta* | 19.48 ± 5.67 | 5.60 ± 1.29 | *df* = 238, t = 26.15, p < 0.001 |
| *Chalybion bengalense* | - | 4.03 ± 1.44 | - |
| *Halictus acrocephalus* | 33.88 ± 12.35 | 3.61 ± 1.38 | *df* = 238, t = 26.68, p < 0.001 |
| *Megachile monticola* | 5.82 ± 2.48 | 3.74 ± 0.97 | *df* = 238, t = 8.56, p < 0.001 |
| *Polistes tenebricosus* | - | 4.08 ± 1.48 | - |
| *Pseudapis oxybeloides* | 31.17 ± 10.92 | 3.38 ± 1.27 | *df* = 238, t = 27.71, p < 0.001 |
| *Scolia soror* | - | 4.32 ± 1.48 | - |
| *Tetragonula iridipennis* | 37.57 ± 12.98 | - | - |
| *Thyreus nitidulus* | 5.77 ± 2.49 | 3.60 ± 1.06 | *df* = 238, t = 8.78, p < 0.001 |
| *Xylocopa aestuans* | - | 2.87 ± 0.79 | - |
| *Xylocopa amethystina* | 3.30 ± 0.89 | 2.47 ± 0.61 | *df* = 238, t = 8.40, p < 0.001 |
| *Xylocopa fenestrata* | - | 2.59 ± 0.87 | - |
| *Xylocopa latipes* | - | 2.76 ± 0.82 | - |
| Lepidoptera |  |  |  |
| *Eretmocera impactella* | - | - | - |
| *Utetheisa pulchella* | - | - | - |

Values are given in mean ± standard deviation.
