# Supplementary material for: Floral visitors of sesame (Sesamum indicum L.): Elucidating their nectar-robbing behaviour and impacts on the plant reproduction
Source: PLoS One. 2024 Apr 18;19(4):e0300398. doi: 10.1371/journal.pone.0300398 (PMC11025750; doi:10.1371/journal.pone.0300398)
Supplement: S5 Table — (DOCX) [file pone.0300398.s007.docx]

**S5 Table.** Percentages of specialized nectar and mixed foragers were recorded for non-robbers and robbers.

| Visitor | Task allocating categories | Percentages of nectar and mixed foragers recorded for non-robbers and robbers | | Statistical analysis |
| --- | --- | --- | --- | --- |
|  |  | Non-robbers | Robbers |  |
| *Apis cerana* | Nectar forager | 59 ± 6.58 | 75.50 ± 5.99 | *df* = 18, t = -5.86, *p* < 0.001 |
|  | Mixed forager | 41 ± 6.58 | 24.50 ± 5.99 | *df* = 18, t = 5.86, *p* < 0.001 |
| *Apis dorsata* | Nectar forager | 60.50 ± 8.32 | 78 ± 9.19 | *df* = 18, t = -4.46, *p* < 0.001 |
|  | Mixed forager | 39.50 ± 8.32 | 22 ± 9.19 | *df* = 18, t = 4.46, *p* < 0.001 |
| *Apis florea* | Nectar forager | 58.50 ± 6.69 | 76.50 ± 5.80 | *df* = 18, t = -6.43, *p* < 0.001 |
|  | Mixed forager | 41.50 ± 6.69 | 23.50 ± 5.80 | *df* = 18, t = 6.43, *p* < 0.001 |

Values are given as mean ± standard deviation.
